# Fibroblasts as a Biological Marker for Curative Resection in Pancreatic Ductal Adenocarcinoma

**DOI:** 10.3390/ijms21113890

**Published:** 2020-05-29

**Authors:** Eriko Katsuta, Omar M. Rashid, Kazuaki Takabe

**Affiliations:** 1 Department of Surgical Oncology, Roswell Park Comprehensive Cancer Center, Buffalo, NY 14263, USA; eriko.katsuta@roswellpark.org; 2 Holy Cross Hospital Michael and Dianne Bienes Comprehensive Cancer Center, Fort Lauderdale, FL 33308, USA; omar.rashid@holy-cross.com; 3 Department of Surgery, Massachusetts General Hospital, Boston, MA 02114, USA; 4 Department of Surgery, University of Miami Miller School of Medicine, Miami, FL 33136, USA; 5 Department of Surgery, Nova Southeastern University School of Medicine, Fort Lauderdale, FL 33328, USA; 6 Department of Surgery, University at Buffalo Jacobs School of Medicine and Biomedical Sciences, The State University of New York, Buffalo, NY 14203, USA; 7 Department of Breast Surgery and Oncology, Tokyo Medical University, Tokyo 160-8402, Japan; 8 Department of Surgery, Yokohama City University, Yokohama 236-0004, Japan; 9 Department of Surgery, Niigata University Graduate School of Medical and Dental Sciences, Niigata 951-8510, Japan; 10 Department of Breast Surgery, Fukushima Medical University School of Medicine, Fukushima 960-1295, Japan

**Keywords:** fibroblast, pancreatic cancer, residual tumor, vascularity

## Abstract

Achievement of microscopic tumor clearance (R0) after pancreatic ductal adenocarcinoma (PDAC) surgery is determined by cancer biology rather than operative technique. Fibroblasts are known to play pro-cancer roles; however, a small subset was recently found to play anti-cancer roles. Therefore, we hypothesized that intratumor fibroblasts contribute to curative resection and a better survival of PDAC. Utilizing a large, publicly available PDAC cohort, we found that fibroblast composition was associated with R0 curative resection. A high amount of fibroblasts in PDACs was significantly associated with a higher amount of mature vessels, but not with blood angiogenesis. A high amount of fibroblasts was also associated with a higher infiltration of anti-cancer immune cells, such as CD8+ T-cells and dendritic cells, together with higher inflammatory signaling, including IL2/STAT5 and IL6/JAK/STAT3 signaling. Further, the fibroblast composition was inversely associated with cancer cell composition in the bulk tumor, along with an inverse association with proliferative characteristics, such as MYC signaling and glycolysis. The patients with high-fibroblast PDACs showed an improved prognosis. In conclusion, we found that PDACs with high fibroblasts were associated with a higher R0 resection rate, resulting in a better prognosis. These findings may be due to less aggressive biology with a higher vascularity and anti-cancer immunity, and a low cancer cell component.

## 1. Introduction

Pancreatic ductal adenocarcinoma (PDAC) is the fourth most common cause of cancer-related death in the USA [1], and its 5-year survival rate is as low as 10% according to the Surveillance, Epidemiology, and End Results (SEER) database. However, it is also known that PDAC patients who had no residual cancer present after undergoing curative surgery resulted in better survival compared to others. Tumor resection with microscopic tumor clearance (R0; ≥ 1 mm circumferential margin) is critical for these patients [2]. Patients who achieved R0 resection demonstrated improved survival compared to those with microscopic tumor infiltration (R1; < 1 mm circumferential margin) or macroscopic residual tumor (R2; cancer at the margin) [2].

It should be noted that the failure to achieve negative margin (i.e., R1/2) resection is not considered to be due to a failure in surgical technique, but rather is thought to be due to the aggressive and diffuse growth pattern of PDAC. Some have reported a prognosis for PDAC patients with residual tumor present after resection (R1/2) similar to that of palliative chemoradiotherapy without resection [3]. Currently, the indications for PDAC surgical resection are determined by a consensus of anatomic resectability criteria designed to select tumors for surgical treatment using multimodality imaging [4]. However, there is increasing evidence that the biological behavior of the cancer plays a critical role in achieving complete curative resection [4], acting as a biological marker which may predict R0 resection, rather than relying on the classic anatomic criteria alone.

Pancreatic fibrosis is one of the parameters associated with a better prognosis [5]. Historically, this has been thought to be due to a lower incidence of post-pancreatectomy pancreatic fistula during the perioperative course of recovery [5,6], and few studies have evaluated its contribution to achieving R0 resection. PDAC contains a large amount of fibroblasts. Numerous studies demonstrated pro-cancer roles for fibroblasts in PDAC by remodeling the tumor microenvironment, resulting in cancer progression and chemo-resistance [7]. On the other hand, it has been also reported in recent studies that fibroblasts play anti-cancer roles as well [8]. The PDAC fibroblasts with different roles are considered to be of different subtypes. It has been reported that there are two subtypes of fibroblasts in PDAC based on specific markers [7]. As an activated fibroblast marker, α-smooth muscle actin (αSMA) is the standard marker for the activated fibroblast. On the other hand, fibroblast activation protein-α (FAPα) is used to identify the pro-tumorigenic fibroblast subset [7]. The combination of these two markers is proposed to possibly distinguish anti-cancer (αSMA+/FAPα−) from pro-cancer (αSMA−/FAPα+) fibroblasts. We previously found that PDACs rich in mature vessels have a high anti-cancer immunity, including high CD8+ T-cell and γδ T-cells and low regulatory T-cell infiltration, resulting in a better prognosis [9]. Therefore, we hypothesized that intra-tumoral fibroblasts are associated with tumor vascularity and thus contribute to achieving curative R0 resection of PDAC.

To this end, we employed a large PDAC cohort, The Cancer Genome Atlas (TCGA), to evaluate whether intra-tumoral fibroblasts were associated with curative resection, utilizing a computational algorithm of tumor component cell fraction estimation. In addition, the biology of fibroblast-rich PDAC was investigated.

## 2. Results

### 2.1. PDAC with High Fibroblasts Associates with Higher R0 Resection

**Figure 1:**
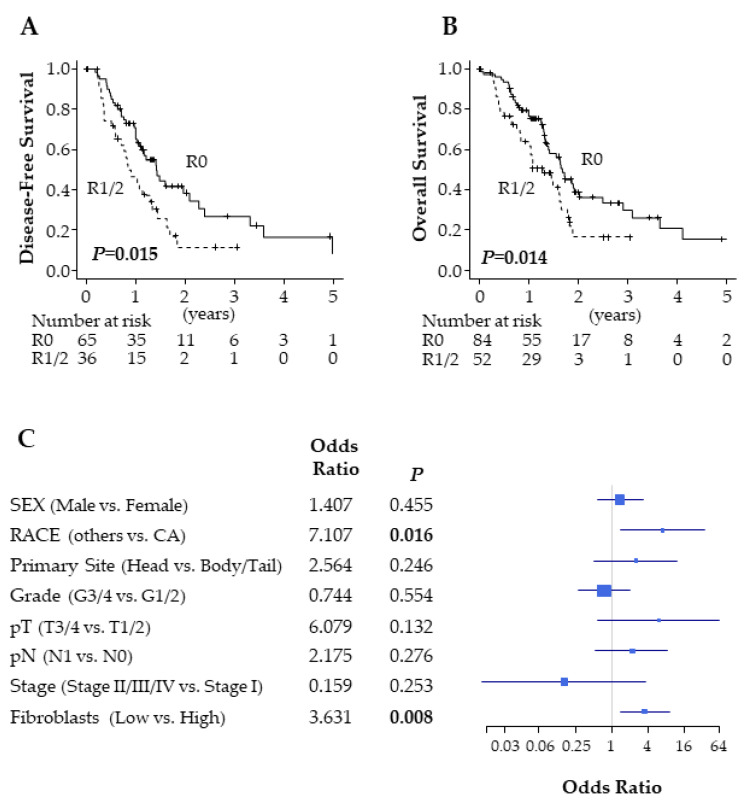
Residual tumor status in pancreatic ductal adenocarcinoma (PDAC). (**A**) Disease-free survival comparing R0 and R1/2 in The Cancer Genome Atlas (TCGA) PDAC cohort. (**B**) Overall survival comparing R0 and R1/2 in TCGA PDAC cohort. (**C**) Forest plot of odds ratio for R1/2 resection. R0; *n* = 84 and R1/2; *n* = 52. CA: Caucasian American.

Achievement of R0 pancreatic resection is a known factor for a better prognosis in PDAC; however, some claim that its determination is not universal [10]. Thus, we examined whether R0 correlated with improved outcomes in TCGA PDAC cohort. Out of 147 PDAC patients, 84 patients were pathologically diagnosed as having no residual tumor status (R0), and the remaining 52 patients were diagnosed as having residual tumor present (R1/2). As expected, the patients after R0 resection had a significantly better disease-free survival (DFS) (*p* = 0.015) and overall survival (OS) (*p* = 0.014) compared with after R1/2 resection (Figure 1A,B). This is in agreement with previous reports [2,10]. Multivariate analysis revealed that only Caucasian American (*p* = 0.016) and high fibroblast PDACs (*p* = 0.008) were significantly associated with R0 resection (Figure 1C).

### 2.2. PDACs Contain a High Amount of Fibroblasts

**Figure 2:**
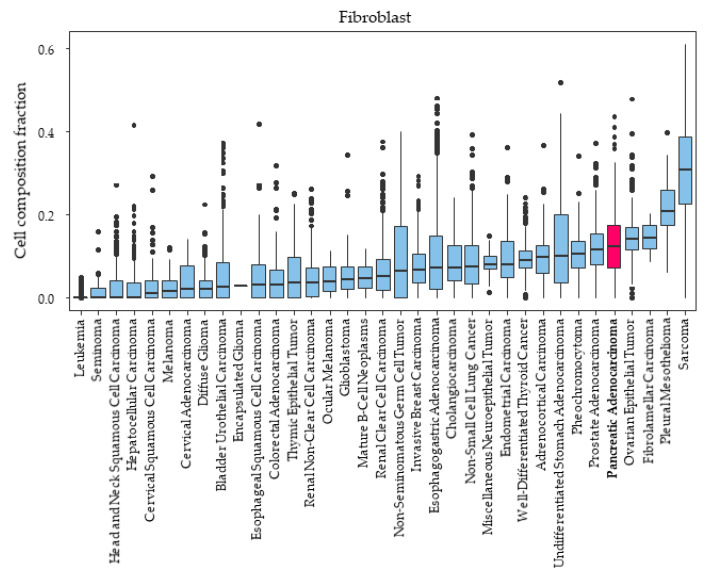
Tumor-infiltrating fibroblast fraction among various types of cancers in TCGA.

It was reported that the majority of the bulk tumors of PDACs consist of fibroblasts [8]; thus, the level of fibroblast composition in PDAC compared with other cancers was of interest. We investigated fibroblast composition amount among various types of cancer in TCGA. PDAC was the fifth-highest in fibroblast composition fraction among the various types of cancer in TCGA (Figure 2).

### 2.3. Fibroblast Subtypes in PDAC Are Indistinguishable by Transcriptomic Detection of Surface Markers Alone

**Figure 3:**
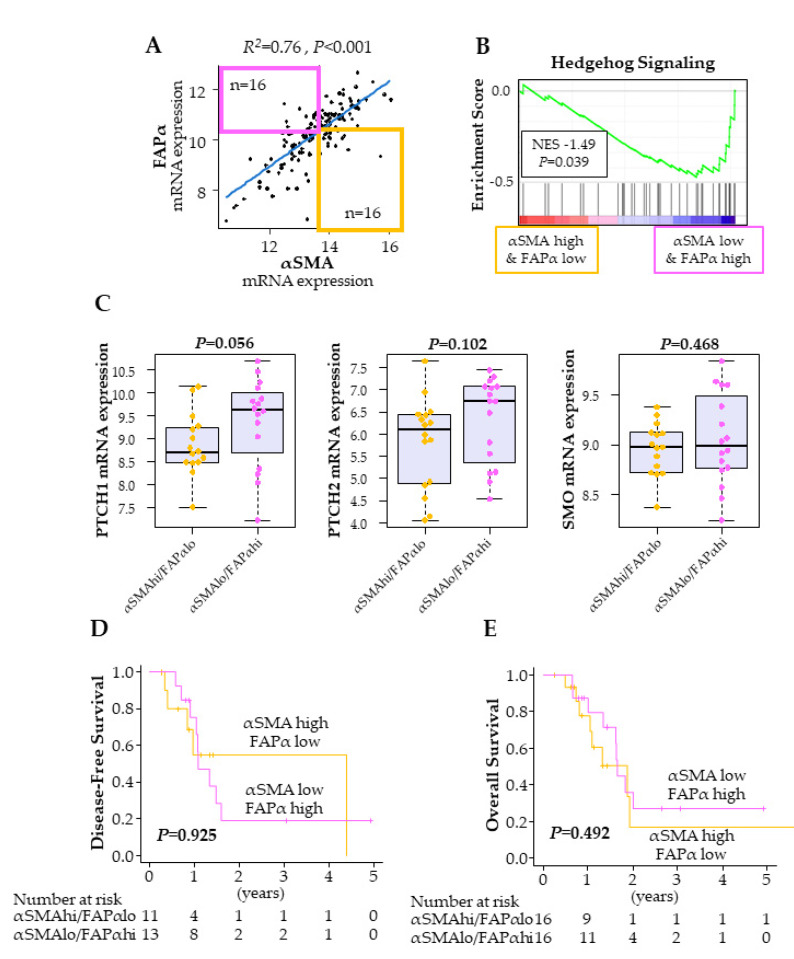
Fibroblast subtype classification of PDAC in TCGA. (**A**) Classification of fibroblast by *αSMA* and *FAPα* expression in PDAC. (**B**) Gene set enrichment analysis (GSEA) between the patients with the two types of fibroblasts. (**C**) Gene expression of Hedgehog signaling regulators comparison between the tumors with two types of fibroblasts. (**D**) Disease-free survival comparison between the patients with the two types of fibroblasts. (**E**) Overall survival comparison between the patients with the two types of fibroblasts.

We investigated whether we could distinguish the two different subtypes of cancer-associated fibroblasts—anti-cancer (αSMA+/FAPα−) and pro-cancer (αSMA−/FAPα+) fibroblasts—by gene expression levels of *αSMA* and *FAPα* in bulk PDAC tumors. The gene expressions of *αSMA* and *FAPα* were highly correlated in TCGA PDAC cohort (R^2^ = 0.76) (Figure 3A), however, there was a small fraction of tumors that differentially expressed these two markers. We defined the cut-off of high vs. low using the median of the gene expression, and we extracted PDACs with *αSMA*-high/*FAPα*-low (*n* = 16) and those with *αSMA*-low/*FAPα*-high (*n* = 16) (Figure 3A). Hedgehog signaling from cancer cells to fibroblasts is thought to play a tumor-suppressive role. Thus, it is believed that anti-cancer (αSMA+/FAPα−) fibroblasts have higher Hedgehog signaling with lower negative regulators, Ptch 1/2, and higher positive regulators, Smo [11]. To our surprise, Hedgehog signaling was negatively associated with *αSMA*-high/*FAPα*-low PDACs in TCGA (Figure 3B). In addition, neither the negative (*Ptch1* and *Ptch2*; *p* = 0.056, *p* = 0.102) nor the positive (*Smo*; *p* = 0.468) regulators of Hedgehog signaling demonstrated any difference between them (Figure 3C). We further evaluated these two subtypes of fibroblasts for any impact on patient survival. There was no significant difference in either DFS (*p* = 0.925) or OS (*p* = 0.492) between *αSMA*-high/*FAPα*-low and *αSMA*-low/*FAPα*-high groups (Figure 3D,E). We also performed an analysis using markers *CD248* and *ITGA8*, which were reported as markers to distinguish lung fibroblast subtypes [12], and there was also no significant survival difference (Figure S1). Given these results that failed to reproduce those of previous studies, we concluded that fibroblast subtypes were indistinguishable when utilizing the gene expressions of fibroblast surface markers in PDAC bulk tumors alone.

### 2.4. Clinicopathological Demographics Are Similar between Fibroblast High and Low PDAC

Given the above results, which failed to differentiate between fibroblast subtypes, we investigated the fibroblast role in total. The patients were divided into high and low fibroblast tumor groups using median cutoff. The pathological findings of aggressive features, such as lymphovascular invasion, tended to be lower (*p* = 0.088) and chronic pancreatitis was higher (*p* = 0.060) in the patients with high fibroblasts, but neither was statistically significant (Table 1). Other patient demographics examined were not significantly different between these two groups, including age, sex, race, primary site, pathological grade, perineural invasion, and AJCC staging, including pT and pN categories (Table 1).

### 2.5. PDACs with High Fibroblasts Associate with a High Vascularity

**Figure 4:**
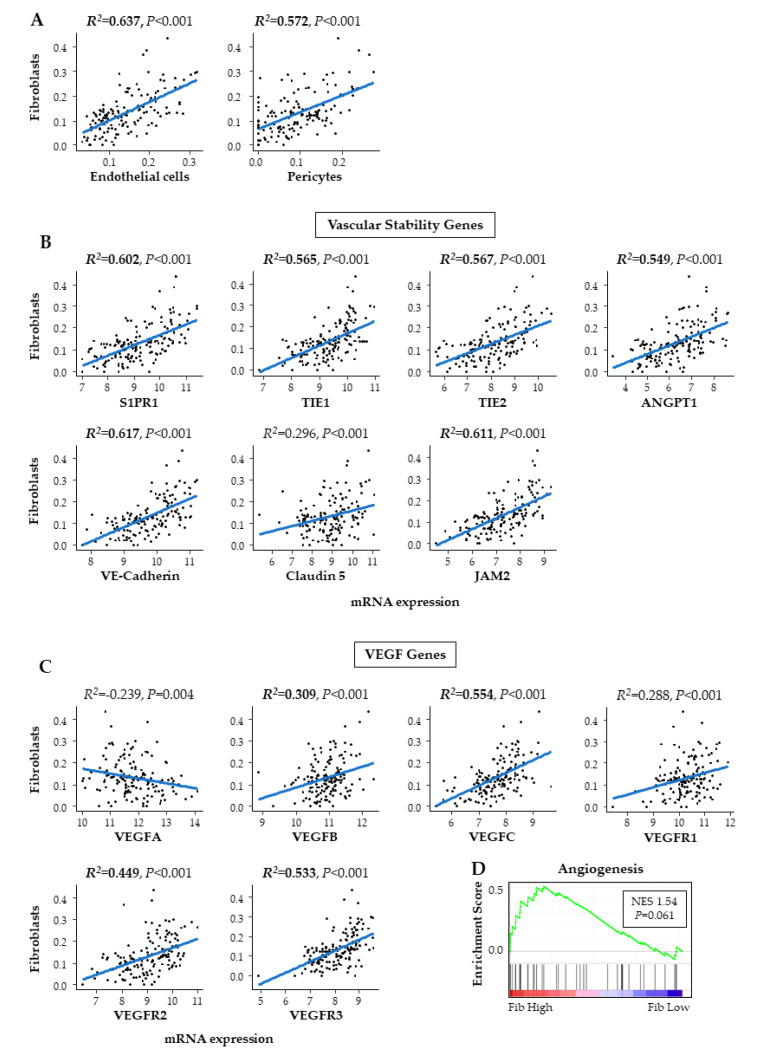
Fibroblasts and vascularity of PDAC in TCGA. (**A**) Correlation plot of fibroblasts and cells of vessel components. (**B**) Correlation plot of fibroblasts and vascular stability genes. (**C**) Correlation plot of fibroblasts and vascular endothelial growth factor (VEGF) genes. (**D**) GSEA comparing high and low fibroblast tumors.

Based on our hypothesis, in which fibroblasts correlated with tumor vascularity, we then analyzed the association of fibroblasts with vascularity in PDAC. The cell composition fraction of the endothelial cells, which are a component of the vascular wall, was moderately correlated with fibroblasts (R^2^ = 0.637) (Figure 4A). Furthermore, the pericytes, which wrap around endothelial cells and stabilize and mature them [13], were also moderately correlated with fibroblasts (R^2^ = 0.572) (Figure 4A). In agreement with this, six out of seven vascular stability markers that are expressed in the mature vessels were correlated with fibroblasts, including *sphingosine-1-phosphate receptor 1* (*S1PR1*; R^2^ = 0.637), *TIE1* (R^2^ = 0.565), *TIE2* (R^2^ = 0.567), *Angiopoietin 1* (*ANGPT1*; R^2^ = 0.549), *VE-cadherin* (R^2^ = 0.617), and *JAM2* (R^2^ = 0.611) (Figure 4B). On the other hand, *vascular endothelial growth factor-A* (*VEGFA*), which is a major player in blood angiogenesis, and its receptor *VEGFR1*, showed no correlation with fibroblasts (R^2^ = −0.239, R^2^ = 0.288, respectively) (Figure 4C). Gene set enrichment analysis (GSEA) also revealed no significant association with angiogenesis in fibroblast high PDACs (Figure 4D). *VEGFB*, of which the role in cancer is still understudied, showed a weak correlation (R^2^ = 0.309) (Figure 4C). *VEGFC*, which plays a role in lymphangiogenesis and its receptors *VEGFR2* and *VEGFR3* were also weakly to moderately correlated with fibroblasts (R^2^ = 0.554, R^2^ = 0.449, R^2^ = 0.533, respectively) (Figure 4C). These findings suggest that high fibroblast PDACs have high vascularity and lymphangiogenesis, but not VEGFA-mediated angiogenesis.

### 2.6. PDACs with High Fibroblasts Associate with High Anti-Cancer Immunity

**Figure 5:**
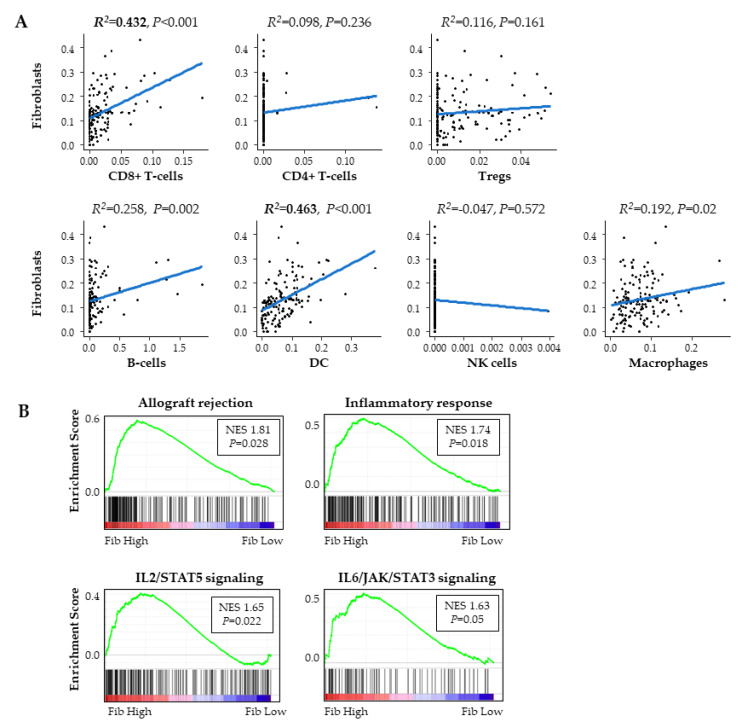
Fibroblasts and immunity of PDAC in TCGA. (**A**) Correlation plot of fibroblasts and infiltrated immune cells. (**B**) GSEA comparing high and low fibroblast tumors. Tregs; regulatory T-cells, DC; dendritic cells, NK cells; natural killer cells.

Fibroblasts in PDAC are also known to be associated with inflammation. Together with the above results that fibroblasts were associated with higher vascularity, and our previous findings that higher vascularity PDAC was associated with higher anti-cancer immunity [9], we further hypothesized that high fibroblast PDACs also have high anti-cancer immunity. As expected, CD8+ T-cells, which participate in cytolytic activity against cancer cells, demonstrated a weak correlation with fibroblasts (R^2^ = 0.432). Dendritic cells, which play a role in antigen presentation, also demonstrated a weak correlation with fibroblasts (R^2^ = 0.463) (Figure 5A). Interestingly, none of the CD4+ T-cells, regulatory T-cells, B-cells, natural killer cells, or macrophages demonstrated a correlation with fibroblasts either. GSEA revealed that high fibroblast PDACs correlated with inflammation-related gene sets, including allograft rejection, inflammatory response, IL2/STAT5 signaling, and IL6/STAT3 signaling (Figure 5B). These findings suggest that fibroblasts correlated with a higher anti-cancer immunity.

### 2.7. PDACs with High Fibroblasts Negatively Associate with Tumoral Cancer Cell Composition and Cancer Proliferation

**Figure 6:**
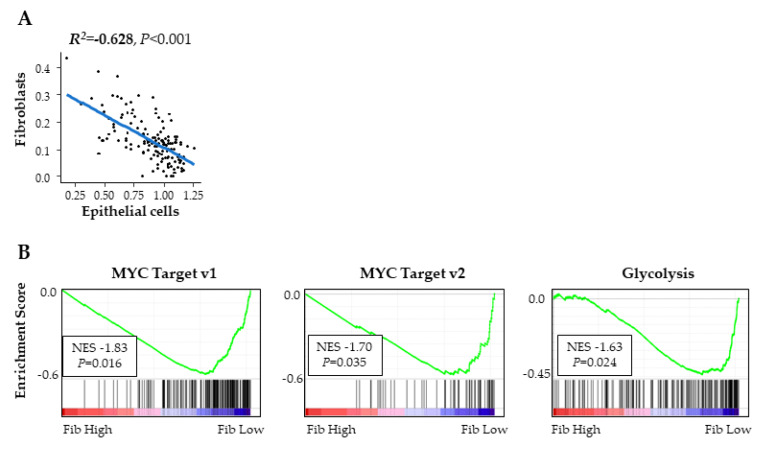
Fibroblasts and cancer cells in PDACs in TCGA. (**A**) Correlation plot of fibroblasts and intratumor epithelial cells. (**B**) GSEA comparing high and low fibroblast tumors.

It has been experimentally demonstrated that low vascularity PDAC tumors contained more cancer cells, and thus correlated with aggressive characteristics [11]. Together with our result that high fibroblast PDACs had higher vascularity, we hypothesized that fibroblast amount inversely associates with the amount of cancer cells present in the tumor. As expected, epithelial cells, which mainly reflect cancer cells, negatively correlated with fibroblasts (R^2^ = −0.628) (Figure 6A). Furthermore, MYC targets, which is one of the most powerful oncogenes and promotes cancer cell proliferation [12], and glycolysis, which promotes cell proliferation, enhances cell survival, and is the metabolic pathway preferred by cancer cells as per the Werburg effect [13], were all negatively associated with fibroblasts (Figure 6B). These findings further imply that high fibroblast PDACs have a lower composition of cancer cells with less cancer proliferation.

### 2.8. PDACs with High Fibroblasts Associate with a Better Prognosis

**Figure 7:**
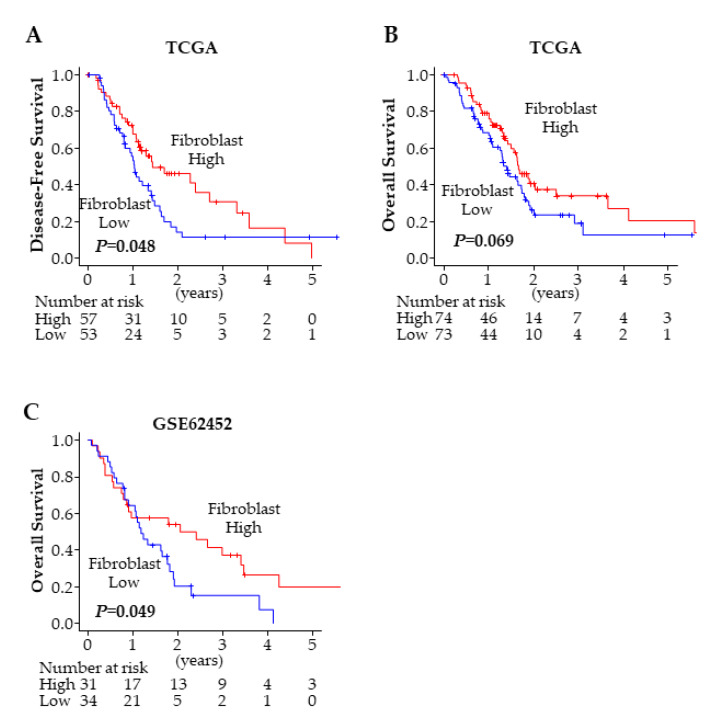
Fibroblast and PDAC prognosis. (**A**) Disease-free survival comparing high and low fibroblast tumors in TCGA PDAC cohort. (**B**) Overall survival comparing high and low fibroblast tumors in TCGA PDAC cohort. (**C**) Overall Survival comparing high and low fibroblast tumors in GSE62452 cohort.

Finally, we investigated the impact of fibroblasts on PDAC patient prognosis. In agreement with the aforementioned results, the patients with high fibroblast PDACs showed significantly better DFS (*p* = 0.048) (Figure 7A), and trended toward a better OS (*p* = 0.069) (Figure 7B). The result was validated in another cohort, GSE62452 [14], in which the patients with high fibroblast tumors (*p* = 0.049) showed significantly better OS in PDAC (Figure 7C). These findings imply that high fibroblast PDACs demonstrated less invasive characteristics, were more likely to achieve an R0 resection, and thus correlated with a better prognosis.

## 3. Discussion

In the current study, we demonstrated that fibroblast composition was associated with R0 curative resection in PDAC, which is one of most fibroblast-rich cancers. We were unable to distinguish the fibroblast subtypes through transcriptomic analysis of surface markers alone in TCGA cohort. High fibroblast PDACs correlated with higher mature blood and lymphatic vascularity, inflammation and anti-cancer immunity, as well as a lower tumoral composition of cancer cells with less cell proliferation. The patients with high fibroblast PDACs demonstrated improved DFS.

Complete curative resection that achieves microscopic tumor clearance (R0) is the only chance to cure PDAC; therefore, it is a predictor of prolonged survival when compared with microscopic or macroscopic remnant tumors after pancreatectomy (R1/R2) [2,15,16]. It is a common misconception among non-surgeons that tumor remains after PDAC resection because of poor surgical technique. Rather, it is well known that the aggressive biology and invasiveness of PDAC resulted in the majority of cases with positive margins (R1/2) [2]. Since PDAC has highly invasive characteristics, R0 resection is often not achieved. Interestingly, in the current study, we demonstrated that there were no significant differences in R status by such characteristics of tumor aggressiveness as pathological grade, lymphovascular invasion, and by advanced stage. Instead, it was a higher tumoral fibroblast composition alone that was associated with curative R0 resection, which is in agreement with the notion that cancer biology dictates complete resectability.

Fibroblasts have been demonstrated to play a pro-cancer role in PDAC; in particular, there have been numerous reports of its role in chemo-resistance [17,18]. It has been demonstrated that fibroblasts physically block chemotherapy agent access to cancer cells, resulting in chemo-resistance. Thus, fibroblast-depleting therapies have been tested; however, the results showed a very small survival benefit in a clinical trial [19], and even led to more aggressive behavior in a pre-clinical study [20]. In contrast, recent studies demonstrated that there is another subtype of fibroblasts which play a tumor-suppressive role [7]. In the current study, we were unable to reproduce the characteristics of fibroblast subtypes using *αSMA* and *FAPα* gene expressions. This may be partially due to the involvement of more complex markers to distinguish these fibroblast subtypes or simply that the transcriptomic signature of these two markers were not specific enough for a bulk tumor. However, interestingly, regardless of subtype classification, we found that PDAC with a higher fibroblast composition on the whole was associated with a higher composition of vessels and immune cells.

Interestingly, higher fibroblast PDACs trended to have a lower lymphovascular invasion rate. This seems somewhat contradictory to the common belief that lymphovascular invasion is associated with high blood and lymphatic vascularity. On the other hand, we recently reported that higher lymphovascular invasion was associated with neither blood nor lymphatic vessel density, but instead with aggressive phenotypes such as a high proliferation marker and a worse prognosis in breast cancer [21]. Our current findings support the notion that lymphovascular invasion reflects aggressive cancer biology, rather than the amount of vascularity.

We previously found that PDACs with mature vessels have a higher anti-cancer immunity, resulting in a better prognosis [9]. In this study, we also found that high fibroblast PDACs showed high vascularity and high anti-cancer immunity. In addition, it is reported that IL2 signaling activates fibroblasts that enhance inflammatory signaling, such as IL6/JAK/STAT3 signaling [7], which is consistent with our findings. In contrast, it has been reported that vascularity is negatively associated with tumor cancer cell composition [22]. Our finding that high fibroblast PDACs negatively associated with cancer cell composition implicates an important link between PDAC components, such as cancer cells, fibroblasts, and vessels. Taken together, high fibroblast PDACs have high vascularity, high anti-cancer immunity and a lower cancer cell composition. Furthermore, the combination of these components was associated with PDAC patient prognosis. Our findings provide new insight into patients with high fibroblast PDAC, that they have a higher possibility of achieving curative resection. Therefore, preoperative assessment of tumor fibroblast levels may become a clinical parameter for evaluating resectability of PDAC cases in the future.

There are limitations in this study. First, the current study analyses were based only on the gene expression of the surgically-resected primary tumor in TCGA cohort. PDAC patients who are treated surgically account for less than 20%. In addition, our results were focused only on the role of fibroblasts in surgically-removed PDACs. Thus, the role in unresectable and metastatic settings, as well as under chemotherapy, may be different. Furthermore, our study is limited in that it was conducted by a bioinformatics approach alone. Immunohistochemistry and flowcytometry are the standard techniques to analyze tumor cell composition; however, we were unable to conduct confirmation studies using these methods due to a lack of access to patient samples. In addition, in order to explore the PDAC fibroblast role further, in vitro and in vivo experimental approaches are needed.

In conclusion, we found that high fibroblast PDACs were associated with a high composition of mature blood and lymphatic vascularity, high anti-cancer immunity, a lower composition of cancer cells and less cell proliferation, and a higher likelihood to achieve microscopic tumor clearance (R0), which resulted in a better prognosis.

## 4. Materials and Methods

### 4.1. Data Acquisition and Patient Classification

The Pancreatic cancer cohort of TCGA was downloaded through cBioPortal [23,24] and used as previously described [25,26]. There are 154 pancreatic cancer patients in TCGA. Among them, there are 147 patients pathologically diagnosed as PDAC and mRNA data from RNA sequence are present. The patients were classified in fibroblast high and low groups using the median cutoff. Lymphovascular invasion, perineural invasion, and chronic pancreatitis information were obtained from each pathology report in cBioPortal. As a validation cohort, we utilized GSE62452, in which there are 65 PDAC patients with gene expression and survival information from the Gene Enrichment Omnibus [14]. The patients were classified as high more than 0 and low for 0 in fibroblast in GSE62452.

### 4.2. Cell Composition Fraction Estimation

A computational algorithm, xCell, was used to estimate the cell composition of a tumor from its gene expression profiles [27]. Data were downloaded through xCell website (https://xcell.ucsf.edu/).

### 4.3. Gene Set Enrichment Analyses (GSEA)

GSEA was performed using Hallmark gene sets with software provided by the Broad Institute (http://software.broadinstitute.org/gsea/index.jsp), as we described previously [28,29].

### 4.4. Statistical Analysis

Factors associated with R0 were analyzed by logistic regression. Gene expression differences were analyzed using the Wilcoxon test, the survival differences were analyzed using Kaplan-Meier curves with log-rank test, and the clinicopathological demographics were compared using the Fisher exact test. Two-sided *p* < 0.05 was considered statistically significant for all tests. All statistical analyses were performed using R software (http:///www.r-project.org/) and Bioconductor (http://bioconductor.org/).

## Figures and Tables

**Table 1:** Patient demographics comparing high and low fibroblast PDACs in TCGA.

	Fibroblast			
	High (*n* = 74)	Low (*n* = 73)		*p*
**Age** (y.o.)	64.2 ± 10.1	65.3 ± 11.6		0.552
**Sex**				
Female	36	32		0.621
Male	38	41		
**Race**				
Caucasian	67	61		0.184
Others	5	10		
**Primary site**				
Head	63	61		0.818
Body/Tail	10	11		
**Tumor size** (cm)	3.71 ± 1.26	3.79 ± 1.48		0.743
**Grade**				
G1/2	53	52		>0.999
G3/4	21	21		
**LVI**				
Negative	25	16		0.088
Positive	38	49		
**PNI**				
Negative	12	5		0.118
Positive	56	63		
**Chronic Pancreatitis**				
Negative	41	52		0.060
Positive	33	21		
**pT**				
pT1/2	10	10		>0.999
pT3/4	64	62		
**pN**				
pN0	20	17		0.705
pN1	54	55		
**Stage**				
Stage I	6	6		>0.999
Stage II/III/IV	68	66		

## Supplementary Materials

Supplementary materials can be found at https://www.mdpi.com/1422-0067/21/11/3890/s1, Figure S1: Fibroblast subtype classification of PDAC in TCGA. (A) Classification of fibroblast by *CD248* and *ITGA8* expression in PDAC. (B) Disease-free survival comparison between the patients with the two types of fibroblasts. (C) Overall survival comparison between the patients with the two types of fibroblasts.

## Abbreviations

PDAC	Pancreatic ductal adenocarcinoma
TCGA	The Cancer Genome Atlas
DFS	Disease-free survival
OS	Overall survival
αSMA	α-smooth muscle actin
FAPα	fibroblast activation protein-α
S1PR	Sphingosine-1-phospate receptor
ANGPT	Angiopoietin
VEGF	Vascular endothelial growth factor
GSEA	Gene set enrichment analysis
