# Supplementary material for: Fibroblasts as a Biological Marker for Curative Resection in Pancreatic Ductal Adenocarcinoma
Source: Int J Mol Sci. 2020 May 29;21(11):3890. doi: 10.3390/ijms21113890 (PMC7312973; doi:10.3390/ijms21113890)
Supplement: Supplementary file 1 [file ijms-21-03890-s001.pdf]

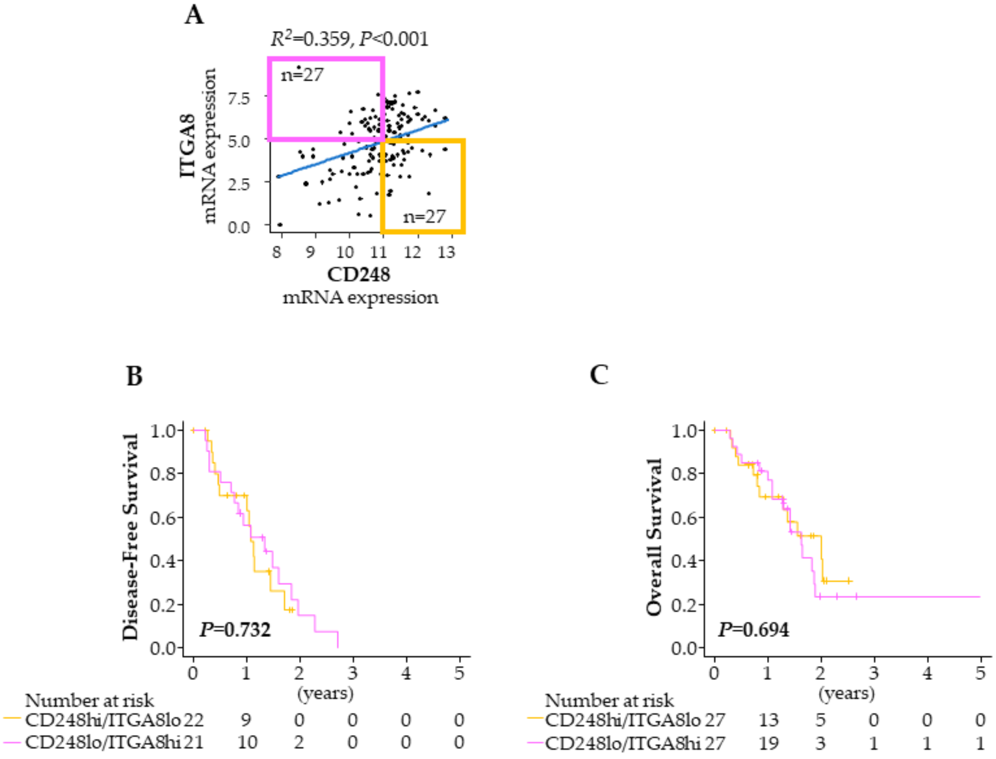

Figure S1. Fibroblast subtype classification of PDAC in TCGA. (**A**) Classification of fibroblast by *CD248* and *ITGA8* expression in PDAC. (**B**) Disease-free survival comparison between the patients with the two types of fibroblasts. (**C**) Overall survival comparison between the patients with the two types of fibroblasts.
